# Supplementary material for: A Gnotobiotic Mouse Model with Divergent Equol-Producing Phenotypes: Potential for Determining Microbial-Driven Health Impacts of Soy Isoflavone Daidzein
Source: Nutrients. 2024 Apr 6;16(7):1079. doi: 10.3390/nu16071079 (PMC11013052; doi:10.3390/nu16071079)
Supplement: Supplementary file 1 [file nutrients-16-01079-s001.zip › nutrients-2926743-supplementary.pdf]

Supplemental Table S1. Bacterial culture media and incubation times. The following broth and agar were used for the growth of each strain in this study. All strains were grown anaerobically at 37 °C. Agar incubation requires an additional 1–2 days to achieve optimal colony growth.

**Species**	**Culture Broth**	**Agar**	**Broth Incubation Time**
Bacteroides caccae	Anaerobic Basal Broth (ABB, DSMZ Medium 1203a)	Brucella Blood Agar (BBA, Thermo Scientific (Cat. No. BD 211086) with 5% blood)	12–24 hours
Bacteroides thetaiotaomicron	ABB	BBA	12–24 hours
Bacteroides uniformis	ABB	BBA	36–48 hours
Roseburia intestinalis	Yeast Casitone Fatty Acid (YCFA) broth	YCFA agar	12–24 hours
Faecalibacterium duncaniae	Reinforced Clostridia Media (RCM, Thermo Scientific (Cat. No. CM0149B)) broth	RCM agar	12–24 hours
Agathobacter rectalis	PGY (DSMZ Medium 104)	BBA	36–48 hours
Coprococcus comes	ABB	BBA	12–24 hours
Akkermansia muciniphila	Anaerobic Brain Heart Infusion broth (A-BHI, ATCC Medium 2187) + 0.4% Mucin	Tryptic Soy Agar (TSA) + 5% Blood (ATCC Medium 260)	36–48 hours
Providencia stuartii	Nutrient Broth (NB, ATCC Medium 3)	TSA + 5% Blood	12–24 hours
Collinsella aerofaciens	ABB	BBA or RCM	12–24 hours
Adlercreutzia equolifaciens	Wilkins-Chagrin (WC, Thermo Scientific (Cat. No. CM0643B)) broth	WC agar	48–72 hours
